# Phytochemistry and Biological Effects of the *Juglans regia* “*Sorrento*” Walnut Husk Extract on Human Keratinocyte Cells

**DOI:** 10.3390/antiox14121385

**Published:** 2025-11-21

**Authors:** Giulia Vergine, Michela Ottolini, Giuseppe E. De Benedetto, Simona Bettini, Francesca Baldassarre, Daniele Vergara, Giuseppe Ciccarella

**Affiliations:** 1 National Interuniversity Consortium of Materials Science and Technology (INSTM), Via G. Giusti 9, 50121 Florence, Italy; giulia.vergine@unisalento.it; 2 Department of Biological and Environmental Sciences and Technologies (DiSTeBA), University of Salento, 73100 Lecce, Italy; michela.ottolini@unisalento.it; 3 Department of Cultural Heritage, University of Salento, Via D. Birago 64, 73100 Lecce, Italy; giuseppe.debenedetto@unisalento.it; 4 Department of Biological and Environmental Sciences and Technologies (DiSTeBA), University of Salento & UdR INSTM Salento, 73100 Lecce, Italy; simona.bettini@unisalento.it (S.B.); giuseppe.ciccarella@unisalento.it (G.C.); 5 Institute of Nanotechnology, Consiglio Nazionale Delle Ricerche, CNR NANOTEC, Via Monteroni, 73100 Lecce, Italy

**Keywords:** walnut husk, UHPLC–Q-Orbitrap HRMS, phytochemicals, antioxidant, HaCat, epithelial homeostasis

## Abstract

Plants are a valuable source of natural compounds with diverse applications. Recently, increased attention has focused on waste products from the agricultural industry, including walnut husk. Given its potential as a sustainable source of bioactives, this work characterizes the alcoholic *Juglans regia* “*Sorrento*” walnut husk extract (WHE). The extract’s phenolic content, antioxidant activity, and phytochemical composition were evaluated using spectrophotometry and UHPLC-HRMS-based untargeted metabolomics analysis. WHE exhibited a high total phenolic content (TPC = 1.45 ± 0.03 mg GAE/g dry extract) and a rich profile of phenolic acids, flavonoids, and tannins. Given this composition, WHE’s biological activity was further tested in an in vitro human keratinocyte (HaCaT) model. At the concentration of 10 μg/mL, WHE showed no cytotoxicity and displayed significant antioxidant properties by modulating detoxifying proteins such as Nrf2. WHE also influenced mitochondrial metabolism, increased maximum respiration, preserved barrier integrity, and activated pathways for epithelial homeostasis. Overall, this study highlights the bioactivity of the *J. regia* “*Sorrento*” walnut husk extract. These findings support the valorization of walnut husk as a sustainable source of bioactives for dermatological and cosmetic products.

## 1. Introduction

The skin serves as the anatomical and physiological boundary between the internal and external environments of the human body. Within its various layers, including the epidermis, dermis, and subcutaneous tissue, many cell types respond to environmental changes by triggering physiological reactions to chemical and physical agents. Maintaining skin homeostasis is crucial, as its alteration may result in the development of various skin disorders.

Plant extracts have long been valued for their potential in dermatology and cosmetics due to their antioxidant properties and ability to modulate intracellular signaling pathways [1]. Among these, walnuts represent a rich source of bioactive phytochemicals, including alkaloids, flavonoids, quinones, and phenolic compounds. The walnut fruit consists of three major parts—the edible kernel, the shell, and the husk—each containing distinct bioactive compounds with potential health benefits [2]. The kernel accounts for about 50% of the total fruit weight and is the only edible part, leading to a large amount of agricultural waste generated annually. However, the non-edible portions exhibit relevant biological properties and can be repurposed in several applications such as biofuel production, wastewater treatment, or natural dye synthesis, in line with circular economy principles [3].

The green husk, representing 20–45% of the fruit’s weight depending on the variety [4,5], is a major by-product. While traditionally used for producing walnut liqueur, it often becomes an industrial waste with disposal challenges. Its potential reuse as a source of organic acids and phenolic compounds offers opportunities for sustainable valorization. The phytochemical composition of walnut husks is known to vary with geographical location, climate, and soil conditions [5,6,7].

The walnut *Juglans regia* L. (Persian or English walnut), a member of the Juglandaceae family, is the second most cultivated tree nut worldwide [8]. Numerous studies have reported the pharmacological potential of *J. regia* extracts, including antioxidant, anti-inflammatory, antimicrobial, antidiabetic, hepatoprotective, neuroprotective, and wound-healing properties [9,10]. In particular, the walnut husk has been primarily investigated for its antitumor activity in models exploring apoptosis and invasion [11,12]. More recently, *J. regia* extracts have attracted attention for dermatological and cosmetic applications. For example, green husk extract has been proposed as a natural hair dye with strong antimicrobial activity compared to synthetic dyes [13]. Beyond these uses, several studies have highlighted its antioxidant, anti-tyrosinase, wound-healing, and anti-aging potential, as well as its role in UV protection and collagenase/elastase inhibition [8].

Despite promising findings, in vivo evidence remains limited, and further studies are needed to elucidate the mechanisms of action of *J. regia* phytochemicals, optimize formulations, and evaluate their safety for dermatological use, including in aesthetic procedures such as photoepilation (consider adding a reference here if available on *J. regia* skin research).

In Italy, walnut cultivation occurs across several regions, with about 30% of production concentrated in Campania, where the “*Sorrento* walnut” (*J. regia* var. *Sorrento*) is the most common cultivar. This indigenous variety, native to the Sorrento Peninsula, is now widely cultivated throughout Campania. However, limited data exist on the phytochemistry and bioactivity of “*Sorrento* walnut” husk extracts, particularly concerning their potential applications for skin health [4].

The present study aims to investigate the effects of *J. regia* “*Sorrento*” walnut husk extract on signaling pathways involved in epithelial homeostasis and antioxidant defense, using a human keratinocyte cell line. We analyzed the phytochemical composition of an alcoholic husk extract and assessed its toxicity and phototoxicity in response to diode light exposure. We also evaluated its antioxidant activity, combining measurements of intracellular ROS levels with proteomic analysis to explore molecular mechanisms underlying redox balance. Finally, we examined its potential influence on skin barrier integrity and mitochondrial function.

## 2. Materials and Methods

### 2.1. Preparation of Plant Extract

The walnut husk extract (WHE) was provided by the cosmetics farmer Licofarma s.r.l. (Galatina, Lecce, Italy). The starting agro-residues were green husks from *Juglans regia* L. belonging to the “*Sorrento*” *cultivar*. Fresh walnut green husks were roughly chopped using a blender with a blade before the extraction process. The procedure provided a maceration in pure Ethanol (96%, for use in perfumery) for 45 days in a closed container, in the dark and at RT. The solid/solvent (g/g) was 1:2.4. The extract was stored at −20 °C before analyses. The final extract concentration was expressed as mg solid extract/mL recovered extract and it was determined following vacuum–evaporation at 40 °C of an aliquot (10 mL), reconstituting with water to a final volume of 10 mL and measuring the weight after freeze-drying.

### 2.2. Phytochemical Analysis

#### 2.2.1. Spectroscopic Characterization

UV-Vis absorption spectra were recorded using a Cary 5000 Agilent UV-Vis spectrophotometer, Santa Clara, CA, USA. For FT-IR characterization, samples were deposited directly onto the ATR element of a FT-IR ATR spectrometer (Perkin-Elmer Spectrum One, Shelton, CT, USA) by drop-casting; after solvent evaporation, 64 scans were collected per measurement over the spectral range of 4000–600 cm^−1^. Spectroscopic-grade solvents were employed for all measurements. All solutions were prepared in ultrapure water (18 MΩ·cm).

#### 2.2.2. Total Phenolic Content (TPC) Determination

Total phenolic content (TPC) was determined using the Folin–Ciocalteu colorimetric assay. The assay relies on the reduction of the Folin–Ciocalteu reagent by phenolic compounds under alkaline conditions, leading to the formation of a blue chromophore with an absorption maximum at 760 nm. The extract was diluted with Folin–Ciocalteu reagent (MP Biomedicals, Irvine, CA, USA) for 3 min. Subsequently, a sodium carbonate solution (Merck, Rahway, NJ, USA, Na_2_CO_3_, ≥99.5% purity) at 10.75% w/v concentration was added, and the mixture was incubated at room temperature for 40 min in the dark to allow complete color development. Absorbance was measured at 760 nm and compared against a gallic acid (Merck, ≥99% purity) calibration curve (0.20 to 1.60 µg/mL, R^2^ = 0.99).

The results, expressed as milligrams of gallic acid equivalents (GAE) per milliliter of extract (mg GAE/mL), were normalized by the extract concentration to obtain the total phenolic content per milligram of dry extract (mg GAE/mg dry extract). All measurements were performed in triplicate (n = 3), and the results are expressed as mean ± standard deviation (SD). The TPC value for WHE was calculated using the following calibration Equation (1):

(1) $$TPC\;=\;\frac{A_{SAMPLE}\;-\;b}{m}\;\;\;[\frac{mg\;GAE}{mL}]$$

where *A_SAMPLE_* is the absorbance measured at 760 nm for the extract solution, *b* is the y-intercept of the gallic acid calibration curve (Figure S1), *m* is the slope (also known as angular coefficient) of the calibration curve.

#### 2.2.3. U HPLC–Q-Orbitrap HRMS Analysis of the Extract

Extracts were analysed on a Q Exactive Plus mass spectrometer (ThermoFisher Scientific, Inc., Waltham, MA, USA) equipped with a heated electrospray ionization (HESI) probe (ThermoScientific). The equipment was operated in negative and positive ion modes within the *m*/*z* range from 75 to 1125. The mass spectrometer parameters were as follows: spray voltage 1.5 kV (+) and 1.5 kV (−); sheath gas flow rate 48; auxiliary gas flow rate 11; spare gas flow rate 2; capillary temperature 256 °C; aux gas heater temperature 413 °C; S-lens RF level 50. The scan mode was Full MS/dd-MS2 (Top N, N = 1), of which the resolution was respectively 70,000 (Full MS) and 17,500 (dd-MS2). The collision energy was set to 35% in the MS/MS mode. The UPLC was performed on an Ultimate 3000 system (Thermo Fisher Scientific, Dionex, Sunnyvale, CA, USA) comprising a quaternary pump a column thermostat and an autosampler and the extracts (injection volume, 2–5 µL) were separated on a Poroshell 120 EC-C18 column (150 × 2.1 mm, 2.7 µm; Agilent, Santa Clara, CA, USA) maintained at 30 °C. The linear gradient of mobile phases between 0.1% formic acid in water (A)/ 0.1% formic acid in acetonitrile (B) was as follows: 0–1 min, 5% B; 1–15 min, 5–95% B; 15–25 min, 95% B; 25–27 min, 95–5% B. Equilibration time was 5 min. Each extract was filtered through a 0.45 µm filter before injection. Data were recorded and analysed using the Xcalibur software (Version 4.0.27, Thermo Fisher Scientific, Waltham, MA, USA).

The raw data were processed using MS-DIAL 5 (Available online: https://github.com/systemsomicslab accessed on 24 June 2025) with default parameters. In positive ion mode, [M + H]^+^, and [M + H–H_2_O]^+^ were selected as the adduct ion types whereas in negative ion mode the adduct ion types were [M + FA]^−^, [M–H]^−^, and [M–2H]^2−^. MSDial annotation was carried out using ESI(+)-MS/MS from authentic standards (16,232 unique compounds) and Fiehn/Vaniya natural product library (MS/MS Positive 28,347 Records) as ESI(+) databases, ESI(−)-MS/MS from authentic standards (8887 unique compounds) and Fiehn/Vaniya natural product library (MS/MS Negative 11,590 records) as ESI(−) databases all downloaded from https://systemsomicslab.github.io/compms/msdial/main.html accessed on 24 June 2025.

### 2.3. Antioxidant Activity

#### 2.3.1. ABTS Assay

The ABTS^•+^ (2,2′-Azino-bis(3-ethylbenzothiazoline-6-sulfonic acid) diammonium salt; Merck) radical scavenging activity was assessed following a method widely reported in the literature [14], with slight modifications. The ABTS radical cation solution was prepared by mixing equal volumes of a 7 mM ABTS stock solution and a 2.45 mM potassium persulfate solution (K_2_S_2_O_8_, ≥99% purity, Merck) and incubated in the dark at room temperature for 12–16 h to allow radical formation. Before the assay, the ABTS^•+^ solution was diluted with phosphate-buffered saline (PBS, pH 7.4) to obtain an absorbance of 0.70 ± 0.02 at 734 nm. Then, 30 μL of the sample or ascorbic acid standard (≥99% purity, Merck) was added to 1.2 mL of the diluted ABTS^•+^ working solution. After a 5-min incubation period in the dark at room temperature, the absorbance was recorded at 734 nm using a Cary 5000 spectrophotometer. Negative control samples were prepared by replacing the extract with PBS and ascorbic acid as positive (standard) control. The scavenging activity was calculated based on the absorbance values of the sample and control. All measurements were carried out in triplicate (n = 3), and results are presented as mean ± standard deviation (SD).

The antioxidant capacity (AC) was calculated using the following Equation (2):

(2) $$AC\%=\;\left[\frac{Abs\;control-Abs\;sample}{Abs\;control}\right]\times \;100$$

where control is the ABTS^•+^ working solution and sample is the tested extract solution.

#### 2.3.2. DPPH Assay

This protocol provided the neutralization of DPPH-free radicals (DPPH reagent from Sigma Aldrich, Italy). Briefly, 0.5 mL of extract at different concentrations was added to 2.5 mL of 0.1 mM DPPH-methanolic solution and vigorously shaken in the dark at room temperature. The absorbance of samples at 515 nm was measured after 30 min. Ascorbic acid and methanol were used as positive (standard) and negative controls, respectively. The scavenging activity was calculated based on the absorbance values of the sample and control. All measurements were carried out in triplicate (n = 3), and results are presented as mean ± standard deviation (SD). The calculation of antioxidant capacity for the DPPH assay was also performed using Equation (2), as described for the ABTS assay. In this case, the control corresponds to the DPPH-methanolic solution and sample is the tested extract solution.

### 2.4. Cell Lines and Culture Treatments

The HaCaT cell line from human skin keratinocytes was cultured and maintained in Dulbecco’s modified Eagle’s medium (DMEM) and supplemented with 10% Fetal Bovine Serum (FBS), 1% penicillin and streptomycin at 37 °C in a 5% CO_2_ incubator.

For the MTT test, cells were plated at 5 × 10^3^ cells per well in a 96-well plate and allowed to adhere to the plate overnight. After treatment, the culture medium was aspirated, and 100 μL of a medium containing 10 μL of MTT stock solution (5 mg mL−1 tetrazole in phosphate-buffered saline) was added. After two hours of incubation, the MTT solution was removed, and 100 μL of solubilization solution was added to dissolve MTT–formazan crystals. The absorbance of the converted dye was measured at a wavelength of 570 nm using a microplate reader (Multiskan™ FC Microplate Photometer, Thermo Fisher Scientific). The relative cell viability was expressed as a percentage of the untreated control group.

Cells were seeded in 96-well plates at a density of 1 × 10^4^ cells per well and allowed to adhere overnight. Then cells were exposed to a commercial diode laser (Licofarma s.r.l., Galatina, Italy) with a wavelength of 808 nm, in the near-infrared (NIR) spectrum. Irradiation was performed under different parameters: 10 J/cm^2^, 3 Hz, 1 W/cm^2^, 20 J/cm^2^, 3 Hz, 1 W/cm^2^, 40 J/cm^2^, 10 Hz, 1 W/cm^2^. After irradiation, cells were incubated for 24 h and then evaluated for cell viability using the MTT assay.

### 2.5. Hydrogen Peroxide and Peroxidase Assays

Hydrogen peroxide (H_2_O_2_) concentration and peroxidase activity were assessed using the Amplex^®^ Red Hydrogen Peroxide/Peroxidase Assay Kit (Invitrogen, Carlsbad, CA, USA, A22188) according to the manufacturer’s instructions. Absorbance was measured at 560 nm using a Biotek Cytation 5 Cell Imaging Multimode Reader (Agilent Technologies). Positive (10 µM H_2_O_2_ or 2 mU/mL HRP) and negative (buffer only) controls were included in each assay and results were expressed as relative absorbance values compared to controls.

### 2.6. Western Blotting

Cell lysates were extracted using RIPA buffer (Cell Signaling, Danvers, MA, USA), and protein concentration was determined by the Bradford protein assay (Bio-Rad, Hercules, CA, USA). Samples were mixed 1:1 with Laemmli buffer (Sigma Aldrich, St. Louis, MO, USA), boiled for 5 min, and 15 μg of protein was separated by 12% SDS–PAGE and transferred to the Hybond ECL nitrocellulose membrane (GE Healthcare, Chicago, IL, USA). The membranes were blocked for 1 h in milk 5% (PanReacAppliChem, Barcelona, Spain) at room temperature and then probed with the appropriately diluted primary antibodies for 1–2 h at room temperature. After three washes with a solution containing 10 mM Tris, pH 8.0, 150 mM NaCl, 0.5% Tween 20 (TBST solution), blots were incubated with secondary HRP-conjugated antibody for 1 h at room temperature (1:2000 dilution). Blots were subsequently developed using a ChemiDoc Imaging System (Bio-Rad).

Primary antibodies (1:1000 dilution) were OXPHOS Cocktail (Abcam, Cambridge, UK, ab110413). E-cadherin (Santa Cruz Biotechnology, Dallas, TX, USA, sc-8426), γ-catenin (Santa Cruz Biotechnology, sc-514115), GAPDH (Santa Cruz Biotechnology, sc-47724), NDRG1 (Sigma-Aldrich, HPA006881), phospho-NDRG1 (Thr346), Filaggrin (Santa Cruz Biotechnology, sc-66192), (Cell Signaling, #5482), p53 (Santa Cruz Biotechnology, sc-126), phospho-p53 (Ser392) (Santa Cruz Biotechnology, sc-51690), MDM2 (Antibodies, Cambridge, UK, A30069), Nrf2 (Antibodies, A308758), phospho-PKC Substrate (Cell Signaling, #6967). Secondary antibodies (HRP-conjugated) were from Cell Signaling (1:2000 dilution) (anti-rabbit IgG, HRP-linked Antibody #7074; anti-mouse IgG, HRP-linked Antibody #7076).

### 2.7. Seahorse XF Oxygen Consumption Rate (OCR) Assay

To assess mitochondrial respiration in HaCaT cells, the Mito Stress Test Kit (Agilent Technologies, Santa Clara, CA, USA) was employed following the manufacturer’s instructions. The oxygen consumption rate (OCR) was measured using the Seahorse XFp Extracellular Flux Analyser (Agilent Technologies). Cells were seeded into an 8-well Seahorse XFp Cell Culture Miniplate (Agilent Technologies) at a density of 2 × 10^4^ cells per well in complete DMEM and allowed to attach at 37 °C in a humidified incubator with 5% CO_2_. The Mito stress test involves the sequential injection of the following drugs: Oligomycin (1.5 µM), Carbonyl cyanide-p-trifluoromethoxyphenylhydrazone (FCCP) (1.5 µM) and Rotenone/Antimycin A (0.5 µM). Three OCR measurements were obtained under each condition. The OCR data were analysed using Seahorse Analytics software (Version: 1.0.0-783+27062c1b760f71c16b5a6396d21f9ef7747947a5) and are expressed as pmoles of O_2_ per minute (OCR).

## 3. Results

### 3.1. Spectroscopic Characterization of WHE

**Figure 1:**
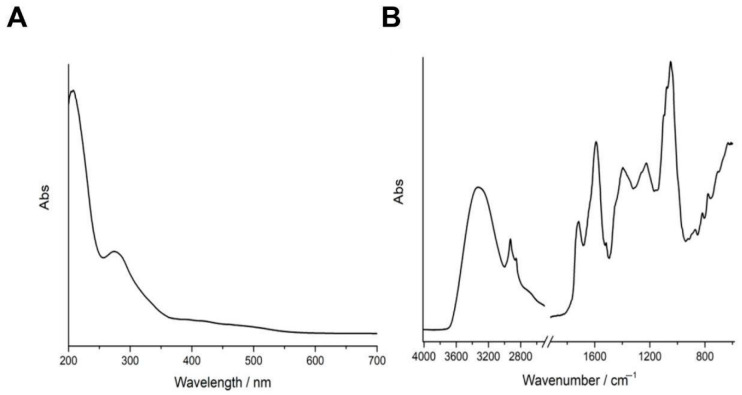
Spectroscopic characterization of WHE. (**A**) UV-Vis and (**B**) FT-IR spectra of WHE solution.

The WHE was first analyzed by UV-Vis and FT-IR spectroscopy. The UV-Vis spectrum (Figure 1A) exhibited a strong absorption band below 300 nm, with a pronounced maximum at approximately 220 nm and a less intense shoulder near 280 nm. The FT-IR spectrum (Figure 1B) showed a broad O–H stretching band in the region 3200–3400 cm^−1^, a C–H stretching vibration near 2920 cm^−1^, and a defined C=O stretching peak at near 1710 cm^−1^. Peaks between 1600 and 1450 cm^−1^ were observed and assigned to aromatic C=C stretching, while the region between 1200 and 900 cm^−1^.

The total phenolic content (TPC) of the WHE was quantitatively determined using the Folin–Ciocalteu colorimetric assay, which is a widely adopted method for evaluating polyphenol concentration in *Juglans regia* L. matrices. The TPC value for WHE was calculated using the calibration Equation (1). Results (μg/mL) were normalized by dividing by the extract concentration (13 mg/mL) to express TPC as mg GAE per g of extract. The WHE showed a TPC value of 1.45 ± 0.03 mg GAE/g of dry extract (i.e., 1450 μg GAE/g), equivalent to 18.6 ± 0.4 μg GAE/mL of solution, (Table 1).

### 3.2. Phytochemical Profile by UHPLC–Q-Orbitrap HRMS

In this study, the UPLC system coupled with Q-Orbitrap system was used to analyse the chemical composition of studied “*Sorrento*”. WHE Phytochemicals have been identified on the basis of their retention times, accurate mass measurements and subsequent mass fragmentation data, and by comparing with literature. Their corresponding molecular formulas, *m*/*z* measured values, mass measurement errors (Δ ppm) and retention times are shown in Table 2, including ESI- and ESI+ mass spectrometric analysis modes. Table 2 shows only the most accurate identifications (82 compounds), and all the proposed molecular formulas were estimated with mass error of less than +1.5 ppm for ESI- and more than −0.91 for ESI+. Furthermore, the most abundant compounds are highlighted in bold and are defined as those with normalized peak heights above the average value.

A total of 57 phenolics were identified, including 9 hydroxycinnamic acids and derivates, 7 hydroxybenzoic acids, 7 flavanols, 4 flavanones,1 flavonols, 2 hydroxy-dihydrochalcones, 2 methylated flavonoids, 8 flavonoid-3-O-glycosides, 5 tannins and 3 hydroquinolones (along with 9 other subclasses). Many of these compounds have been previously detected in walnuts as indicated by attached references. As illustrated in Supplementary Table S1, the identified compounds are reported in accordance with their chemical classification.

For what concerns the phenolic acids class, we found an equal division between the two subclasses hydroxycinnamic and hydroxybenzoic acids. Ferulic acid was identified as the only hydroxycinnamic acid with the [M─H^−^ ion (*m*/*z*) 195,065 (peaks 38 and 60), consistent with the literature [14,17,26]. The other hydroxycinnamic acid derivates identified through their fragmentation patterns included: 3-p-coumaroylquinic acid and derivates (peaks 32 and 33), chlorogenic acid (peaks 23 and 24) and its isomer neochlorogenic acid (peaks 34 and 36) [5,17,22]. Instead quinic acid derivates were detected with [M─H]^−^ ion (*m*/*z*) 191,056 (peaks 22 and 25) [17]. In addition, the key intermediate shikimic acid has been detected at [M─H]^−^ ion (*m*/*z*) 173,045 (peak 2) and 5-O-feruloylquinic acid, with the molecular formula C_17_H_20_O_9_, at [M─H]^−^ ion (*m*/*z*) 367,103 (peak 41), with no specific reference in the literature for walnut husks.

Gallic acid, protocatechuic acid, syringic acid, and vanillic acid were reported as the significant hydroxybenzoic acids in different walnut extracts [22]. The consistent content of gallic acid derivatives in walnut husks has been confirmed [5]. Four gallic acid derivates with the *m*/*z* 169,014 were successfully identified at peaks 4, 8, 11 and 13 [17]. 3,4-dihydroxybenzoic acid was identified through the fragmentation patterns of ions *m*/*z* 153,019 (peaks 19 and 37), it is also known as protocatechuic acid or pyrocatechuic acid [20]. Syringic acid was associated at peak 45 with the *m*/*z* 197,045 at RT 9202. To our knowledge, vanillylmandelic acid secondary metabolite has been detected in WHE for the first time, through the fragmentation patterns of [M─H]^−^ ions *m*/*z* 197,045 (peak 14). Finally, among phenolic acids we have identified two coumarin derivatives, esculetin and scopoletin. Esculetin (6,7-dihydroxycoumarin) has been identified with [M─H]^−^ ion (*m*/*z*) 177,019 (peak 400) as just previously detected in *Juglans regia* L. leaves and husks [19,24].

In our WHE, 7 flavanols were detected identifying epicatechin ([M─H]^−^ *m*/*z* 289,071, peaks 35, 39 and [M─H]^+^ *m*/*z* 291,086 peaks 27 and 30), taxifolin ([M─H]^−^ *m*/*z* 303,051 peak 62 and [M─H]^+^ *m*/*z* 305,065 peak 48) and epicatechin gallate derivates ([M─H]^−^ *m*/*z* 441,082 peak 556 and [M─H]^+^ *m*/*z* 443,097 peak 46) as just described for *J. regia* L. husks [5,17]. Naringenin and eriodictyol were previously reported in the flavanones family ant they have been identified respectively with [M─H]^−^ *m*/*z* 271,061 and [M─H]^+^ *m*/*z* 273,075 (peaks 73 and 69) and [M─H]^+^ *m*/*z* 289,07 (peak 655) [17]. Sternbin, also known as 7-O-methyleriodictyol, is a methylated derivative of eriodictyol and it has been reported for the first time in *J. regia* L. husks with molecular formula C_16_H_14_O_6_ and [M─H]^+^ *m*/*z* 303,086 (peak 71). The flavonols quercetin and (+/−)-dihydrokaempferol have been identified respectively at [M─H]^−^ *m*/*z* 301,035 (peak 75) and [M─H]^−^ *m*/*z* 287,056 (peak 67). The presence of quercetin has been reported in several works on *J. regia* L. WHE, instead the presence of dihydrokaempferol has been previously reported for *J. nigra* extracts [17,19,26]. The investigated WHE resulted particularly rich in flavonoid-3-O-glycosides, detecting hyperoside (peak 43), quercetin-3-arabinoside (peak 47), kaempferol 3-O-glucoside (peak 51), myricitrin (peak 55), astilbin (peak 58), quercetin 3-arabinoside (peak 59), quercetin-3-o-beta-d-xylopyranoside (peak 61), and quercetin 3-rhamnoside (peak 63). For all these compounds have been found correspondence in literature for what concern walnut tissues including husk extracts [17,25]. The presence of other flavonoid compounds was also detected. Peaks 68 and 72 have been assigned to molecular formula C_15_H_14_O_5_ identifying 2 beta-D-glucopyranosyl phloretin and phloretin as confirmed by literature [17]. Furthermore, two methylated flavonoids have been detected in walnut husks for the first time. The methylated derivative of luteolin, the luteolin 3′,4′-dimethyl ether, was identified with [M─H]^−^ *m*/*z* 313,071 (peak 76); the compound velutin has been identified with [M─H]^+^ *m*/*z* 315,08 (peak 74).

Tannins content reported in literature has been confirmed by our analysis identifying 1,6-digalloyl-beta-D-glucopyranose ([M─H]^−^ *m*/*z* 483,07 peaks 28 and 31), 1,2,3,6-tetragalloylglucose ([M─H]^−^ *m*/*z* 787,099 peak 53) and ellagic acid ([M─H]^−^ *m*/*z* 300,999 peak 54) [17,23]. At peak 57 has been identified for the first time another tannin as [3,4,5-Trihydroxy-6-[[(E)-3-(4-Hydroxyphenyl)Prop-2-Enoyl]Oxymethyl]Oxan-2-Yl] 3,4,5-Trihydroxybenzoate with [M─H]^−^ *m*/*z* 477,103 and molecular formula C_22_H_22_O_12_.

Finally, three derivatives of 2-hydroxyquinoline were identified among the main phenolic compounds, with [M─H]^−^ *m*/*z* 144,04 (peaks 17 and 21) and [M─H]^+^ *m*/*z* 146,060 (peak 20).

As for other compounds that do not belong to the phenol or polyphenol classes, the presence of organic acids was also confirmed, with L-malic acid (peaks 5 and 12), citric acid (peak 7) and citramalic acid (peak 9) detected, as previously characterised for walnut husks, shells and leaves, including *J. regia* L. and *J. Nigra* [16,18,19]. Our analysis has been identified also ascorbic acid (peak 49), D-pantothenic acid (peaks 15 and 16), amino acids and derivatives as just previously detected in different *J. regia* L. *cultivars* [15]. To our knowledge the arginine derivative octopine ([M─H]^+^ m/a 247,140, peak 64) has not been detected previously in extracts from walnut husks or other parts of the plant, as well as other identified compounds pyrogallin (peak 66, [M─H]^−^ *m*/*z* 203,035) and sayaendoside (peak 52, [M─H]^−^ *m*/*z* 415,160). Finally, sugars, lipids and other components of cells membrane have been identified (peaks 77–82).

### 3.3. Antioxidant Activity of WHE

The antioxidant capacity of WHE was evaluated using ABTS and DPPH radical scavenging assays. In the ABTS assay, WHE exhibited complete inhibition (100%) at concentrations ≥0.167 mg/mL, with the effect plateauing across higher concentrations. In the DPPH assay, the maximum inhibition reached approximately 68% at 0.667 mg/mL (Table 3). At the highest tested concentration (0.667 mg/mL), ascorbic acid, used as a positive control in both assays, showed 100% inhibition.

By extending the tested concentration range to lower values (0–0.04 mg/mL), a more detailed dose–response profile of WHE in the ABTS assay was obtained, as shown in Figure S2. From this curve, the IC_50_ value was determined to be 0.022 mg/mL.

### 3.4. Biological Evaluation of the Walnut Husk Extract

**Figure 2:**
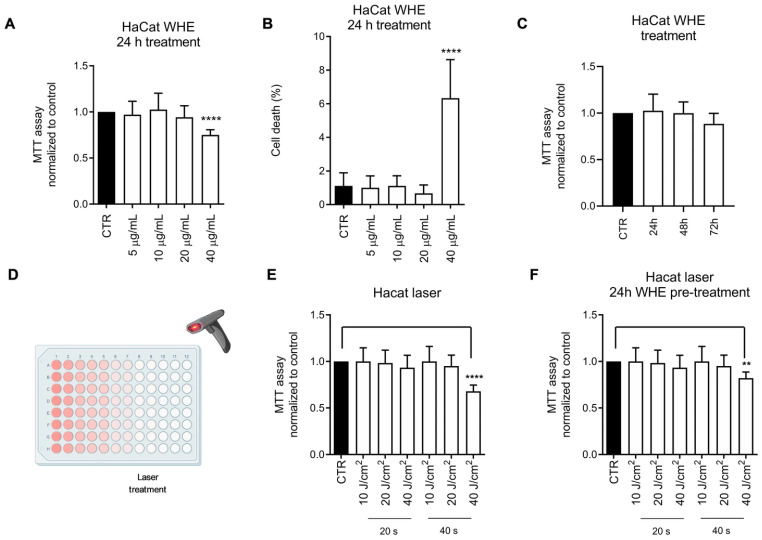
Effect of walnut husk extract on cell viability under standard conditions and after laser treatment. (**A**) MTT assay performed on HaCaT cells exposed to increasing concentration of walnut extract (5–40 μg/mL). (**B**) Cell death obtained performing crystal violet staining. (**C**) Time-course MTT assay at 10 μg/mL extract showing no cytotoxic effect (24–72 h). (**D**) Schematic representation of HaCaT cells irradiated with a diode laser (808 nm) (Created in BioRender. Vergara, D. (2025) https://BioRender.com/uwqc3ic). (**E**,**F**) MTT assay of HaCaT cells after 24 h laser exposure, with or without walnut husk extract pre-treatment. Data represent the means ± SD of three independent experiments. (**) *p* < 0.01; (****), *p* < 0.001.

To evaluate the potential toxic effects of the husk extract on the HaCaT cell line, cells were treated with increasing concentrations of the extract for 24 h. Cell viability was measured using the MTT assay, and dead cells were identified through crystal violet staining. As shown in Figure 2A, a statistically significant reduction in cell viability was observed only at a concentration of 40 μg/mL, accompanied by an increase in the percentage of dead cells (Figure 2B). The effectiveness of pulsed light and/or laser treatment in photoepilation depends on melanin’s ability to absorb light. Any substance capable of absorbing light could cause toxic effects in the skin, related to increased temperature and activation of cellular stressors. To evaluate the potential phototoxic effect of the extract, we assessed the viability of HaCaT cells in response to diode light treatment, either without or after a 24-h pretreatment with the husk extract. As shown in the figure, pretreatment with the WHE husk extract does not induce any phototoxic effect (Figure 2E,F).

**Figure 3:**
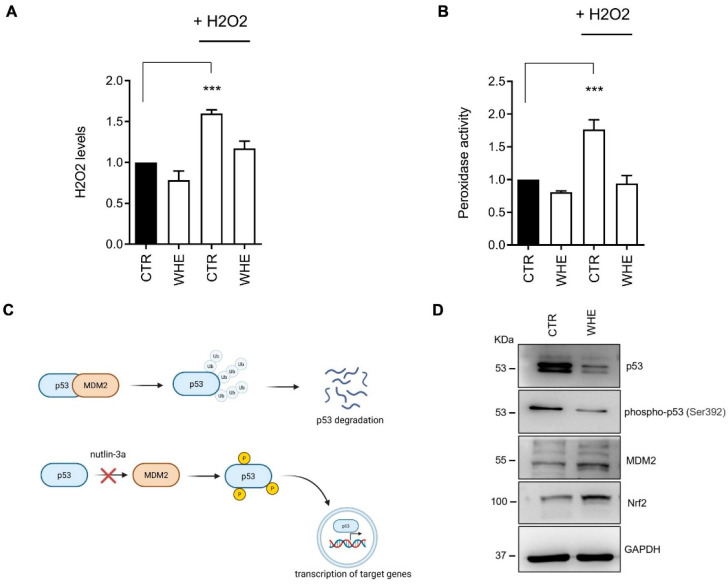
Antioxidant effects of walnut husk extract in HaCaT cells. (**A**) Treatment with the extract (10 µg/µL) reduced intracellular H_2_O_2_ levels induced by exogenous H_2_O_2_. (**B**) Peroxidase activity, which increased in response to H_2_O_2_, was attenuated in the presence of the extract. Data represent the means ± SD of three independent experiments. *p* < 0.01; (***). (**C**) Schematic representation of p53 regulation. (Created in BioRender. Vergara, D. (2025) https://BioRender.com/hfd731n) (**D**) Western blot analysis showing decreased levels of p53 and phosphorylated p53, together with increased MDM2 and Nrf2 expression, supporting the antioxidant role of the extract. GAPDH was used as a loading control. Densitometric quantification of Western blots is reported in Figure S3.

Furthermore, as previously described, the husk extract has been shown to have in vitro antioxidant activity. HaCat cells were treated with 1 mM H_2_O_2_ for 4 h or pretreated with the husk extract at a concentration of 10 μg/mL for 24 h. As shown in Figure 3, treatment with H_2_O_2_ significantly increased intracellular ROS levels in treated cells compared to control cells. Conversely, pretreatment with husk extract prevented the increase in ROS induced by H_2_O_2_. The possible molecular mechanisms responsible for the antioxidant action of the husk extract were investigated through Western blotting, evaluating the expression of intracellular proteins involved in ROS elimination processes. As shown in Figure 3, treatment with husk extract leads to an increase in the levels of the protein Nrf2, a key regulator of the cellular antioxidant response. These effects are achieved through the interaction of Nrf2 with several signaling pathways that regulate cellular processes, including the p53 pathway [28]. The increased expression of p53 coordinates the inactivation of Nrf2 and the activation of an apoptotic program. In cells treated with husk extract, the expression of p53 and its phosphorylated form is reduced, suggesting that the extract acts by modulating the activity of Nrf2 without inducing apoptosis via p53. Intracellular p53 activity is regulated through a feedback loop involving MDM2, a p53-specific E3 ubiquitin ligase. MDM2 is the major cellular antagonist of p53 (Figure 3C). Treatment with husk extract increases MDM2 levels, downregulating p53 expression (Figure 3D).

**Figure 4:**
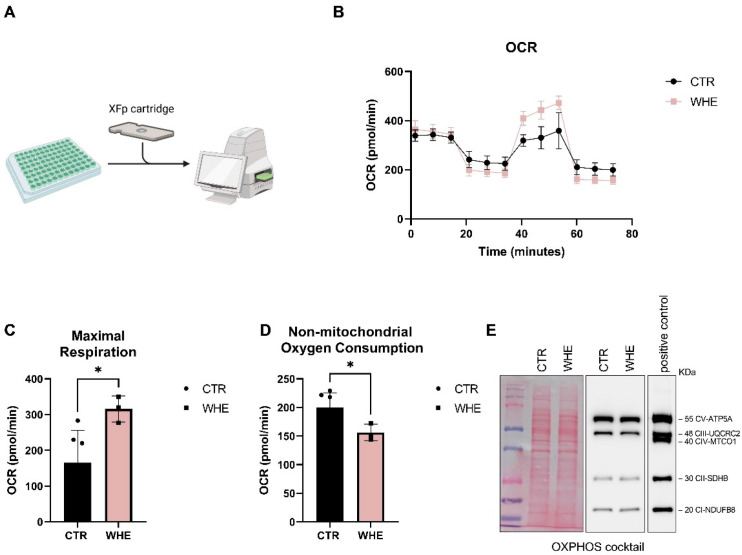
Impact of walnut husk extract (10 μg/mL) on mitochondrial function and bioenergetics in HaCaT cells. (**A**) Schematic representation of Seahorse XF analysis. (Created in BioRender. Vergara, D. (2025) https://BioRender.com/ah0q7dc). (**B**) Oxygen consumption rate (OCR) profile measured by Seahorse XF analysis following sequential injections of oligomycin (1.5 μM), FCCP (1.5 μM), and rotenone/antimycin A (0.5 μM). (**C**,**D**) Quantification of mitochondrial respiration parameters derived from OCR values. Data represent the means ± SD of two independent experiments (CTR = 6 and WHE = 3 replicates). (*) *p* < 0.05. (**E**) Western blot analysis of OXPHOS subunits in control and extract-treated HaCaT cells (10 µg/µL, 24 h). Densitometric quantification of Western blots is reported in Figure S3.

Mitochondria play a key role in maintaining physiological homeostasis in the skin. Alterations in these mitochondria underlie pathological processes in the skin. To assess the effects of husk extract on mitochondrial respiration, we treated HaCat cells with the extract and measured their respiratory capacity using the Seahorse Mito Stress Test. As shown in Figure 4, cells treated with the extract exhibited an increase in maximum mitochondrial capacity (Figure 4C) and a decrease in non-mitochondrial oxygen consumption (Figure 4D) compared to control cells. Since changes in mitochondrial oxidative phosphorylation system (OXPHOS) complexes may affect mitochondrial metabolism, we investigated the expression of OXPHOS complexes by Western blotting. As shown in Figure 4E, the expression of OXPHOS complexes were not significantly affected. Our results indicate that the enhanced respiratory activity in HaCat cells treated with the husk extract is not linked to modification of respiratory proteins.

**Figure 5:**
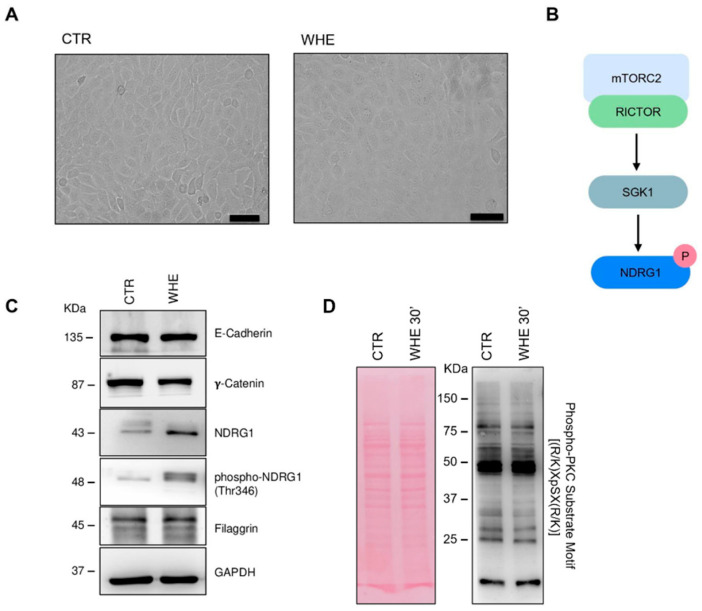
Walnut husk extract preserves epithelial barrier. (**A**) HaCaT cells treated for 24 h with walnut husk extract (10 µg/mL) showed no morphological alterations compared to control. Scale bars 100 μm. (**B**) Schematic representation of the mTORC2 pathway. (**C**) Western blot analysis of junctional proteins (E-cadherin, γ-catenin) and proteins involved in barrier regulation (NDRG1, phospho-NDRG1, Filaggrin) revealed preserved barrier integrity. GAPDH was used as a loading control. (**D**) Effects of walnut husk extract on the expression of phospho-PKC substrate after 30 min of treatment. Ponceau Red used as loading control. Densitometric quantification of Western blots is reported in Figure S3.

Maintaining barrier integrity is essential for regulating skin homeostasis. This process involves a network of molecules and signalling pathways that create a barrier against various agents. Among the proteins involved are junction proteins, differentiation markers, and signalling pathways such as mTORC2 and PKC. Treatment with husk extract has no significant effect on the expression of junction proteins like E-cadherin and γ-catenin (Figure 5C). It also does not change the expression of filaggrin, a protein vital for the proper formation and function of the skin barrier, in particular by assembling the lipid–keratin matrix and ensuring its proper structure and function [29]. PKC is one of the main signaling pathways involved in the regulation of filaggrin expression [30]. In our study, Western blot analysis showed no significant differences in the levels of PKC downstream phosphorylated substrates (Figure 5D). This finding is consistent with the expression data of filaggrin, which also remained unchanged between control and WHE-treated cells (Figure 5C). Furthermore, the extract activates the mTORC2 pathway, which is important in skin morphogenesis and epidermal barrier formation [31], as evidenced by the activation of NDRG1, a marker of this signalling pathway (Figure 5B).

## 4. Discussion

As evidenced by UV–Vis spectroscopy, WHE exhibited strong absorption below 300 nm, with a primary maximum at 220 nm and a shoulder near 280 nm—regions characteristic of π–π* transitions associated with conjugated aromatic systems. These features are typically assigned to simple phenolic acids (e.g., gallic and protocatechuic acid), hydroxycinnamic acid esters (such as chlorogenic and feruloylquinic acid), and condensed flavanols including epicatechin. Additionally, the tailing of the spectrum up to 400 nm may reflect the presence of larger, conjugated structures such as ellagitannins and flavonoid glycosides. The recorded spectral profile is in good agreement with other walnut husk extract [14,32]. FT-IR spectroscopy further corroborated these findings, revealing broad O–H stretching between 3200–3400 cm^−1^, indicative of polyhydroxylated compounds, as well as a sharp C=O stretching band at ~1710 cm^−1^, attributable to carboxylic acids and esters—particularly abundant in hydroxycinnamate–quinic acid conjugates. Peaks in the 1600–1450 cm^−1^ region reflect aromatic C=C ring vibrations, while signals in the 1200–900 cm^−1^ range correspond to C–O stretches of phenolic glycosides, including galloyl esters and flavonoid-3-O-glycosides [5,14,33]. The FT-IR C=O and C-O stretching bands suggests the presence of esterified phenolic acid, such as chlorogenic, feruloylquinic, supporting the next These spectral characteristics are directly supported by HPLC–Q-Orbitrap HRMS analysis, which identified a diverse array of phenolic classes in WHE.

The high total phenolic content (1.4 mg GAE/g dry extract) observed in WHE is consistent with its chromatographic profile, confirming its nature as a polyphenol-rich matrix. The strong response in the Folin–Ciocalteu assay reflects the abundance of hydroxylated aromatic compounds with high reducing capacity. The use of ethanol as a polar protic solvent proved effective for extracting phenolic acids and flavonoids, highlighting the efficiency and environmental compatibility of this extraction method. Overall, the elevated TPC of WHE is both quantitatively significant and qualitatively supported by the identified phenolic constituents.

The UHPLC–Q-Orbitrap HRMS analysis combined with database matching identified 82 compounds in the WHE, mainly phenolic acids (hydroxybenzoic and hydroxycinnamic acids), flavonoids (flavonoid-3-O-glycosides, flavanols, and flavanones), and tannins. These results are consistent with previous findings on *Juglans regia* L. husks as rich sources of phenolic constituents [5,17]. Naphthoquinones, particularly juglone, have been previously described as characteristic compounds of walnut husks compared with other tissues such as pellicle, buds, and bark [5,17]. Medic et al. demonstrated that the majority of naphthoquinones and hydroxycinnamic acids are concentrated in the inner and outer husks after methanol extraction. However, the maceration method applied in this study—favoring longer diffusion and solvent–matrix interaction—appears more efficient for solubilizing medium- and high-polarity compounds such as flavonoid glycosides and hydrolysable tannins, while being less effective for low-polarity constituents like juglone. The absence of juglone in our extract is consistent with its low solubility in hydroalcoholic solvents [7,9] and desirable for safety reasons, since naphthoquinones show cytotoxicity and can induce rare cases of skin irritation or hyperpigmentation [23].

Several biologically active phenolic acids and flavonoids were identified, including compounds not previously reported in walnut husk extracts. Chlorogenic acid derivatives, already described in walnut extracts [5,17,22], were confirmed, supporting their recognized antioxidant, hepatoprotective, antimicrobial, anticancer, and anti-inflammatory properties [34,35]. Shikimic acid (peak 2, Table 2), a key intermediate in chlorogenic acid biosynthesis, was detected for the first time in WHE, although it has been quantified in *J. regia* leaves and decoctions [16,34,35,36]. Among coumarin derivatives, esculetin and scopoletin were identified, confirming previous reports of hydroxycoumarins in walnut tissues [17]. Esculetin (peak 40) exhibits therapeutic effects in atopic dermatitis [23], while scopoletin (peak 50; C_10_H_8_O_4_, [M–H]^+^ 193.049) possesses antifungal properties and was identified in WHE for the first time [37]. Two methylated flavonoids, velutin and luteolin 3′,4′-dimethyl ether (peaks 74 and 76), were also detected for the first time in *J. regia* husk, both known for antioxidant and anti-inflammatory effects. Velutin, in particular, has demonstrated anti-melanogenesis and skin-whitening activity [38,39].

Moreover, 2-hydroxyquinoline was reported here for the first time in walnut extracts, whereas 8-hydroxyquinoline had previously been found in the pellicle and hull of different *Cultivar* [40]. Although rarely described in plant matrices, quinoline and quinazoline alkaloids form a large class of over 600 natural N-based heterocycles [41].

Quantitative analysis of normalized peak heights confirmed phenolic acids and flavonoids as the predominant constituents in WHE. Quercetin and (−)-epicatechin were the main flavonoids [42,43], while ellagic acid was confirmed as the major hydrolysable tannin [23]. The extraction solvent significantly influenced the phytochemical profile: acetone, ethanol, and methanol are reported to yield higher bioactive contents and antioxidant capacities [23]. In particular, 96% ethanol and prolonged extraction times enhanced the recovery of epicatechin, 3-p-coumaroylquinic acid, chlorogenic acid, and gallic acid [42,44]. Additional detected constituents included organic acids (L-malic and citric acid) and the nutritional factor D-(+)-pantothenic acid, which contribute to the extract’s chemical complexity and bioactive potential.

Phenolic compounds, especially flavonoids, are recognized for their antioxidant properties linked to hydrogen-donating capacity. The high phenolic content of WHE suggests strong antioxidant potential, although previous reports on walnut husk activity are inconsistent [7,45,46]. The extended maceration time (45 days) was selected to favor a more complete extraction of phenolic compounds, resulting in an extract with enhanced antioxidant potential. In our study, WHE showed complete ABTS inhibition at low concentrations and moderate DPPH activity, confirming its functional potential. Expanding the tested concentration range allowed a more accurate assessment of the antioxidant profile of WHE, revealing a strong ABTS scavenging activity with an IC_50_ of 0.022 mg/mL. This finding further supports the high antioxidant capacity of the extract obtained after prolonged maceration.

Moreover, it is important to note that the scavenging capacity values obtained by colorimetric methods are not directly comparable with in vivo results. Consequently, to ensure reliable results, these methods must be combined with cellular tests.

Phytochemicals derived from different parts of the *J. regia* are known for their bioactivity on various skin conditions. Many studies demonstrated pharmaceutical and cosmetic uses of *J. regia* extracts through their significant antioxidant activity [8]. Bioactive compounds present in walnut husk extract contribute to its antioxidant properties, supporting its potential use in protecting cells from oxidative stress and related damage. The results of in vitro experiments on the HaCat cell line show that, at the concentrations tested, the extract alone or after phototreatment does not have any toxic effects. The studies demonstrate the extract’s antioxidant activity, which may be linked to the activation of Nrf2. As is common with many secondary metabolites extracted from plants, the extract appears to regulate mitochondrial function by increasing maximum respiration and reducing non-mitochondrial oxygen consumption, although there is no significant increase in the expression of respiratory chain complex proteins. The increase in maximal respiration reflects an enhanced energetic reserve capacity, consistent with improved mitochondrial efficiency rather than increased mitochondrial content. It cannot be excluded that this improvement in mitochondrial efficiency may be related to an activation of signaling pathways involved in the regulation of mitochondrial biogenesis and function, including the mTOR–PGC-1α axis, which plays a key role in controlling oxidative metabolism and mitochondrial turnover [47].

Since non-mitochondrial OCR is largely attributed to oxygen-consuming enzymes such as cyclooxygenases, lipoxygenases, and NADPH oxidases, which are linked to inflammatory processes and considered negative indicators of bioenergetic health [48], its reduction suggests that the treatment may alleviate inflammation-related oxygen consumption. Overall, these effects indicate a protective role in maintaining keratinocyte bioenergetics and homeostasis, consistent with previous evidence linking mitochondrial dysfunction, impaired respiration, and increased oxidative stress to skin ageing [49,50]. mTOR signalling plays a fundamental role in forming the epidermal barrier. Specifically, mTORC2 activity is essential for the phosphorylation of SGK1 and its downstream substrate NDRG1. The function of the NDRG1 protein in epidermal physiology remains incompletely understood. Data from the Human Protein Atlas (https://v19.proteinatlas.org/ENSG00000104419-NDRG1/tissue/skin# accessed on 10 July 2025) confirm its in vivo expression in keratinocytes and other cell types. The increase in phosphorylation following extract treatment can be regarded as a readout of mTORC2 signalling. However, the simultaneous increase in total protein levels does not rule out transcriptional regulation of the gene.

In recent years, plant-derived compounds have attracted the attention of the scientific community for their potential cosmetic applications. Overall, our study highlights the potential biological effects of husk extract in cosmetics, given its significant antioxidant activity and ability to modulate biological processes related to skin ageing. Its potential cosmetic use, however, requires some considerations, including: (i) the possibility of testing the extract’s action on other cell populations (i.e., melanocytes) in three-dimensional environments that can allow for the evaluation of autocrine and paracrine interactions between cell types; (ii) the possibility of using specific formulations that protect the extract from degradation phenomena that could limit its biological effect. In this perspective, future studies could also explore nanoformulation approaches (e.g., liposomes or polymeric nanoparticles) to improve the extract’s stability, extend its shelf-life, and preserve its biological activity for potential inclusion in advanced cosmeceutical preparations [51].

## 5. Conclusions

The findings of this study provide insight into the phytochemical profile, antioxidant, and biological activity of “*Sorrento*” *J. regia* walnut husk extract. Both spectroscopic characterization and UHPLC–HRMS analysis confirmed the presence of a rich composition of flavonoids, tannins, and other metabolites. In vitro experiments conducted on a keratinocyte model show the absence of potential toxic effects and the biological potential of the extract. In detail, tests carried out on HaCat cells confirm the antioxidant capacity of the WHE, demonstrate an increase in mitochondrial respiration and suggest a protective role toward inflammation-related oxygen consumption. Overall, these results highlight *J. regia* walnut husk as a natural source of bioactive compounds for potential cosmetic development.

## Figures and Tables

**Table 1:** TPC of walnut husk extracts measured by Folin–Ciocalteu assay. Values are mean ± SD (*n* = 3).

Extract	Extract Concentration (mg/mL)	GAE Detected at 0.02 mg/mL (μg/mL)	TPC (mg GAE/g Dry Extract)	TPC (μg GAE/mL of Extract)
WHE	13.3	0.028 ± 0.02	1.45 ± 0.03	18.6 ± 0.4

**Table 2:** Characterization of the components from WHE by UHPLC–Q-Orbitrap HRMS.

N.	RT (min)	Compound Identification	Ione Mode	(*m*/*z*)	Δ ppm	Formula	Classification	Ref.
1	1.576	Adenine	[M─H]^−^	134.047	1.079	C_5_H_5_N_5_	6-Aminopurines	[15]
2	1.616	**Shikimic Acid**	[M─H]^−^	173.045	1.105	C_7_H_10_O_5_	Phenolic acids	[16]
3	1.712	D-Glyceric Acid	[M─H]^−^	105.019	1.057	C_3_H_6_O_4_	Sugar Acids and Derivatives	-
4	1.812	Gallic Acid isomer	[M─H]^−^	169.014	1.105	C_7_H_6_O_5_	Phenolic acids (hydroxybenzoic acids)	[5,17]
5	1.982	**L-Malic Acid**	[M─H]^−^	133.014	1.073	C_4_H_6_O_5_	Beta Hydroxy Acids and Derivatives	[17,18,19]
6	2.043	Arabinofuranosyluracil	[M─H]^−^	243.062	1.139	C_9_H_12_N_2_O_6_	Pyrimidine Nucleosides	-
7	2.071	**Citric Acid**	[M─H]^−^	191.01	1.104	C_6_H_8_O_7_	Tricarboxylic Acids and Derivatives	[17,18,19]
8	2.097	Gallic Acid isomer	[M─H]^−^	169.014	1.105	C_7_H_6_O_5_	Phenolic acids (hydroxybenzoic acids)	[5,17]
9	2.259	Citramalic Acid	[M─H]^−^	147.029	1.084	C_5_H_8_O_5_	Hydroxy Fatty Acids	-
10	2.299	**Tyramine**	[M─H]^+^	138.091	−0.912	C_8_H_11_NO	Phenethylamines	-
11	2.836	**Gallic Acid isomer**	[M─H]^−^	169.01425	1.105	C_7_H_6_O_5_	Phenolic acids (hydroxybenzoic acids)	[5,17]
12	3.022	L-Malic Acid Derivate 2	[M─H]^−^	133.014	1.073	C_4_H_6_O_5_	Beta Hydroxy Acids and Derivatives	[17,18,19]
13	3.083	**Gallic Acid hexoside**	[M─H]^−^	169.014	1.105	C_7_H_6_O_5_	Phenolic acids (hydroxybenzoic acids)	[5,17]
14	3.554	Vanillylmandelic Acid	[M─H]^−^	197.045	1.127	C_9_H_10_O_5_	Methoxyphenols	-
15	4.331	D-Pantothenic Acid	[M─H]^−^	218.103	1.132	C_9_H_17_NO_5_	Secondary Alcohols	[15]
16	4.479	D-Pantothenic Acid isomer	[M─H]^−^	218.103	1.132	C_9_H_17_NO_5_	Secondary Alcohols	[15]
17	4.69	2-Hydroxyquinoline isomer	[M─H]^−^	144.045	1.113	C_9_H_7_NO	Hydroquinolones	-
18	4.922	**Hyacinthacine**	[M─H]^+^	220.117	−0.883	C_9_H_17_NO_5_	Alkaloid	[15]
19	6.388	Pyrocatechuic Acid	[M─H]^−^	153.019	1.101	C_7_H_6_O_4_	Phenolic acids (hydroxybenzoic acids)	[20]
20	6.4	**2-Hydroxyquinoline**	[M─H]^+^	146.060	−0.902	C_9_H_7_NO	Hydroquinolones	-
21	6.482	2-Hydroxyquinoline	[M─H]^−^	144.045	1.113	C_9_H_7_NO	Hydroquinolones	-
22	6.636	Quinic Acid	[M─H]^−^	191.056	1.11	C_7_H_12_O_6_	Phenolic acids (Quinic Acids and Derivatives)	[17]
23	6.848	Chlorogenic Acid	[M─H]^+^	355.102	−0.794	C_16_H_18_O_9_	Phenolic acids (hydroxycinnamic acid derivative)	[21]
24	6.98	**Chlorogenic Acid isomer**	[M─H]^+^	355.102	−0.794	C_16_H_18_O_9_	Phenolic acids (hydroxycinnamic acid derivative)	[21]
25	7.218	Quinic Acid Derivate 2	[M─H]^−^	191.056	1.11	C_7_H_12_O_6_	Phenolic acids (Quinic Acids and Derivatives)	[17]
26	7.304	**Tryptophan**	[M─H]^+^	205.097	−0.872	C_11_H_12_N_2_O_2_	Indolyl carboxylic acids and derivatives	[15]
27	7.436	**(-)-Epicatechin**	[M─H]^+^	291.086	−0.818	C_15_H_14_O_6_	Flavanols (Catechins)	[17,22]
28	7.663	1,6-Digalloyl-Beta-D-Glucopyranose	[M─H]^−^	483.078	1.286	C_20_H_20_O_14_	Tannins	[17,23]
29	7.904	Dihydrocoumaroyl Hexoside	[M─H]^−^	327.108	1.206	C_15_H_20_O_8_	Phenolic acids	-
30	7.916	**(-)-Epicatechin isomer**	[M─H]^+^	291.086	−0.818	C_15_H_14_O_6_	Flavanols (Catechins)	[17,22]
31	8.025	1,6-bis-O-galloyl-beta-D-glucose	[M─H]^−^	483.078	1.286	C_20_H_20_O_14_	Tannins	[17,23]
32	8.032	**Coumaroylquinic Acid**	[M─H]^−^	337.092	1.216	C_16_H_18_O_8_	Phenolic acids (hydroxycinnamic acid derivative)	[5,17,22]
33	8.172	**Coumaroylquinic Acid isomer**	[M─H]^−^	337.092	1.216	C_16_H_18_O_8_	Phenolic acids (hydroxycinnamic acid derivative)	[5,17,22]
34	8.212	Neochlorogenic Acid	[M─H]^−^	353.087	1.221	C_16_H_18_O_9_	Phenolic acids (hydroxycinnamic acid derivative)	[22]
35	8.305	**Catechin**	[M─H]^−^	289.071	1.196	C_15_H_14_O_6_	Flavanols (Catechins)	[17,22]
36	8.337	Neochlorogenic Acid isomer	[M─H]^−^	353.087	1.221	C_16_H_18_O_9_	Phenolic acids (hydroxycinnamic acid derivative)	[22]
37	8.381	Pyrocatechuic Acid isomer	[M─H]^−^	153.019	1.101	C_7_H_6_O_4_	Phenolic acids (hydroxybenzoic acids)	[20]
38	8.418	Ferulic Acid	[M─H]^+^	195.065	−0.881	C_10_H_10_O_4_	Phenolic acids (Hydroxycinnamic acids)	[17]
39	8.445	**Catechin isomer**	[M─H]^−^	289.071	1.196	C_15_H_14_O_6_	Flavanols (Catechins)	[17,22]
40	8.572	Esculetin	[M─H]^−^	177.019	1.122	C_9_H_6_O_4_	Phenolic acids (coumarin derivative)	[19,24]
41	8.58	5-O-Feruloylquinic Acid	[M─H]^−^	367.103	1.232	C_17_H_20_O_9_	Phenolic acids (hydroxycinnamic acid derivative)	-
42	8.918	Epiaxifolin	[M─H]^+^	305.065	−0.814	C_15_H_12_O_7_	Flavonoids (Flavanols)	[22]
43	9.192	**Hyperoside**	[M─H]^+^	465.102	−0.727	C_21_H_20_O_12_	Flavonoids (Flavonoid-3-O-glycosides)	[17,25]
44	9.194	(4S,5Z,6S)-4-(2-Methoxy-2-Oxoethyl)-5-[2-[(E)-3-Phenylprop-2-Enoyl]Oxyethylidene]-6-[(2S,3R,4S,5S,6R)-3,4,5-Trihydroxy-6-(Hydroxymethyl)Oxan-2-Yl]Oxy-4H-Pyran-3-Carboxylic Acid	[M─H]^+^	303.049	−0.814	C_15_H_10_O_7_	Null	-
45	9.202	Syringic Acid	[M─H]^−^	197.045	1.127	C_9_H_10_O_5_	Phenolic acids (hydroxybenzoic acids)	[20]
46	9.374	Epicatechin Gallate	[M─H]^+^	443.097	−0.725	C_22_H_18_O_10_	Flavonoids (Flavanols)	[17]
47	9.441	Quercetin-3-Arabinoside	[M─H]^+^	435.092	−0.742	C_20_H_18_O_11_	Flavonoids (Flavonoid-3-O-glycosides)	[17]
48	9.539	**Taxifolin**	[M─H]^+^	305.065	−0.814	C_15_H_12_O_7_	Flavonoids (Flavanols)	[22]
49	9.645	Ascorbic Acid	[M─H]^−^	175.024	1.099	C_6_H_8_O_6_	Butenolides	[15]
50	9.662	Scopoletin	[M─H]^+^	193.049	−0.881	C_10_H_8_O_4_	Phenolic acids (coumarin derivative)	[17]
51	9.693	**Kaempferol 3-O-glucoside**	[M─H]^+^	449.107	−0.731	C_21_H_20_O_11_	Flavonoids (Flavonoid-3-O-glycosides)	[17]
52	9.863	Sayaendoside	[M─H]^−^	415.160	1.259	C_19_H_28_O_10_	O-Glycosyl Compounds	-
53	9.946	1,2,3,6-Tetragalloylglucose	[M─H]^−^	787.099	1.473	C_34_H_28_O_22_	Tannins	[17]
54	10.241	**Ellagic Acid**	[M─H]^−^	300.999	1.194	C_14_H_6_O_8_	Hydrolyzable Tannins	[17,23]
55	10.246	**Myricitrin**	[M─H]^−^	463.088	1.288	C_21_H_20_O_12_	Flavonoids (Flavonoid-3-O-Glycosides)	[17,25]
56	10.434	Catechin Gallate	[M─H]^−^	441.082	1.29	C_22_H_18_O_10_	Flavonoids (Flavanols)	[17]
57	10.496	[3,4,5-Trihydroxy-6-[[(E)-3-(4-Hydroxyphenyl)Prop-2-Enoyl]Oxymethyl]Oxan-2-Yl] 3,4,5-Trihydroxybenzoate	[M─H]^−^	477.103	1.299	C_22_H_22_O_12_	Tannins	-
58	10.569	Astilbin	[M─H]^−^	449.108	1.284	C_21_H_22_O_11_	Flavonoids (Flavonoid-3-O-Glycosides)	[17,25]
59	10.569	Quercetin 3-arabinoside	[M─H]^−^	433.077	1.273	C_20_H_18_O_11_	Flavonoids (Flavonoid-3-O-Glycosides)	[17,25]
60	10.728	4-Hydroxy-3-methoxycinnamic acid isomer	[M─H]^−^	193.050	1.133	C_10_H_10_O_4_	Phenolic acids (Hydroxycinnamic Acids)	[17]
61	10.825	Quercetin-3-o-beta-d-xylopyranoside	[M─H]^−^	433.077	1.273	C_20_H_18_O_11_	Flavonoids (Flavonoid-3-O-Glycosides)	[17,25]
62	10.875	Taxifolin isomer	[M─H]^−^	303.051	1.201	C_15_H_12_O_7_	Flavonoids (Flavanols)	[22]
63	10.922	**Quercitrin Derivate 2**	[M─H]^−^	447.093	1.284	C_21_H_20_O_11_	Flavonoids (Flavonoid-3-O-Glycosides)	[17]
64	11.111	Octopine	[M─H]^+^	247.140	−0.876	C_9_H_18_N_4_O_4_	Arginine and derivatives	-
65	11.247	Eriodictyol	[M─H]^+^	289.07	−0.818	C_15_H_12_O_6_	Flavonoids (Flavanones)	[17]
66	11.77	Pyrogallin	[M─H]^−^	203.035	1.144	C_11_H_8_O_4_	Tropolones	-
67	11.944	(+/-)-Dihydrokaempferol	[M─H]^−^	287.056	1.196	C_15_H_12_O_6_	Flavonoids (Flavonols)	[17,19,26]
68	11.961	2 beta-D-glucopyranosyl Phloretin	[M─H]^+^	275.091	−0.823	C_15_H_14_O_5_	Flavonoids (2′-Hydroxy-dihydrochalcones)	[17]
69	12.121	Naringenin	[M─H]^+^	273.075	−0.823	C_15_H_12_O_5_	Flavonoids (Flavanones)	[17]
70	13.029	**Quercetin**	[M─H]^−^	301.035	1.2	C_15_H_10_O_7_	Flavonoids (Flavonols)	[17]
71	13.079	Sternbin	[M─H]^+^	303.086	−0.808	C_16_H_14_O_6_	Flavonoids (Flavanones)	-
72	13.97	Phloretin	[M─H]^−^	273.076	1.192	C_15_H_14_O_5_	Flavonoids (2′-Hydroxy-Dihydrochalcones)	[17]
73	14.127	Naringenin chalcone	[M─H]^−^	271.061	1.192	C_15_H_12_O_5_	Flavonoids (Flavanones)	[17]
74	14.647	Velutin	[M─H]^+^	315.086	−0.797	C_17_H_14_O_6_	Flavonoids (7-O-methylated flavonoids)	-
75	16.358	Progesterone	[M─H]^+^	315.231	−0.769	C_21_H_30_O_2_	Null	[27]
76	17.605	Luteolin 3′,4′-Dimethyl Ether	[M─H]^−^	313.071	1.218	C_17_H_14_O_6_	Flavonoids (4′-O-Methylated Flavonoids)	[17]
77	18.362	LPE 18:3	[M─H]^−^	474.262	1.293	C_23_H_42_NO_7_P	Lysophosphatidylethanolamine	-
78	19.503	LPI 18:2	[M─H]^−^	595.288	1.356	C_27_H_49_O_12_P	Lysophosphatidylinositol	-
79	19.555	LPE 18:2	[M─H]^−^	476.278	1.293	C_23_H_44_NO_7_P	Lysophosphatidylethanolamine	-
80	20.521	LPE 16:0	[M─H]^−^	452.278	1.272	C_21_H_44_NO_7_P	Lysophosphatidylethanolamine	-
81	21.159	LPE 18:1	[M─H]^−^	478.293	1.294	C_23_H_46_NO_7_P	Lysophosphatidylethanolamine	-
82	22.529	Mannose	[M─H]^−^	179.056	1.1	C_6_H_12_O_6_	Hexoses	[15]

There are only metabolites with normalized peak height ≥ 1 × 10^−4^. The bold font indicates the most abundant compounds referring to the normalized peak height ≥ average value.

**Table 3:** Radical scavenging activity (% inhibition) of the two extracts in ABTS and DPPH assays at increasing concentrations. Values are expressed as mean ± SD (*n* = 3).

Concentration (mg/mL)	ABTS–WHE (% Inhibition)	DPPH–WHE (% Inhibition)
0.667	100 ± 2	68 ± 3
0.550	100 ± 1	66 ± 2
0.333	100 ± 2	60 ± 3
0.167	100 ± 1	64 ± 2
0.083	93 ± 2	52 ± 3
0.040	89 ± 3	38 ± 2

## Data Availability

All experimental data were provided in the manuscript or Supplementary Materials. UHPLC-HRMS data are available on request from authors.

## Supplementary Materials

The following supporting information can be downloaded at https://www.mdpi.com/article/10.3390/antiox14121385/s1. Figure S1. Gallic acid calibration curves for Folin–Ciocalteu colorimetric assay. Figure S2. IC_50_ values of antioxidant activities monitored by ABTS assay. Linear regression curve of ABTS radical scavenging activity of WHE extracts; the red star indicates the calculated IC_50_ value. Figure S3. Densitometric quantification of all proteins analyzed through western blot. Histograms comparing protein expression levels in (A) p53, phospho-p53, MDM2, Nrf2, E-cadherin, γ-catenin, NDRG1, phospho-NDRG1, and filaggrin; and (B) OXPHOS complexes. Protein levels are plotted on a linear scale and expressed as fold change relative to control. Each bar represents the mean ± SD of at least three independent experiments, with values normalized to GAPDH. Graphs were generated using GraphPad Prism (version 9.5). Statistical significance was evaluated by t-test between CTR and WHE. (ns) not significant; (*) *p* < 0.05; (**) *p* < 0.005; (***) *p* < 0.001. Table S1. WHE compounds identified by UHPLC–Q-Orbitrap HRMS based on their chemical classification.

## Abbreviations

The following abbreviations are used in this manuscript:

OCR	Oxygen Consumption Rate
PKC	Protein Kinase C
NDRG1	N-myc downstream regulated 1
TPC	Total phenolic content
UHPLC–HRMS	The ultra-high performance liquid chromatography–high-resolution mass spectrometry system
WHE	Walnut Husk Extract

## References

[1] Gǎlbǎu C.-Ş., Irimie M., Neculau A.E., Dima L., Pogačnik Da Silva L., Vârciu M., Badea M. (2024). The Potential of Plant Extracts Used in Cosmetic Product Applications—Antioxidants Delivery and Mechanism of Actions. Antioxidants.

[2] Mukarram S.A., Wandhekar S.S., Ahmed A.E.M., Pandey V.K., Csaba O., Lajos D., József P., Harsányi E., Bela K. (2024). Exploring the Ecological Implications, Gastronomic Applications, and Nutritional and Therapeutic Potential of *Juglans regia* L. (Green Walnut): A Comprehensive Review. Nutrients.

[3] Jahanban-Esfahlan A., Jahanban-Esfahlan R., Tabibiazar M., Roufegarinejad L., Amarowicz R. (2020). Recent Advances in the Use of Walnut (*Juglans regia* L.) Shell as a Valuable Plant-Based Bio-Sorbent for the Removal of Hazardous Materials. RSC Adv..

[4] Ferrara E., Cice D., Piccolella S., Esposito A., Petriccione M., Pacifico S. (2024). ‘Sorrento’ and ‘Tulare’ Walnut Cultivars: Morphological Traits and Phytochemical Enhancement of Their Shell Waste. Molecules.

[5] Medic A., Jakopic J., Solar A., Hudina M., Veberic R. (2021). Walnut (*J. regia*) Agro-Residues as a Rich Source of Phenolic Compounds. Biology.

[6] Sharma M., Sharma M., Sharma M. (2022). A Comprehensive Review on Ethnobotanical, Medicinal and Nutritional Potential of Walnut (*Juglans regia* L.). Proc. Indian Natl. Sci. Acad..

[7] Jahanban-Esfahlan A., Ostadrahimi A., Tabibiazar M., Amarowicz R. (2019). A Comprehensive Review on the Chemical Constituents and Functional Uses of Walnut (*Juglans* spp.) Husk. Int. J. Mol. Sci..

[8] Adamovic M., Adamovic A., Andjic M., Dimitrijevic J., Zdravkovic N., Kostic O., Pecarski D., Pecarski T., Obradovic D., Tomovic M. (2024). The Botany, Phytochemistry and the Effects of the *Juglans regia* on Healthy and Diseased Skin. Cosmetics.

[9] Delaviz H., Mohammadi J., Ghalamfarsa G., Mohammadi B., Farhadi N. (2017). A Review Study on Phytochemistry and Pharmacology Applications of *Juglans regia* Plant. Pharmacogn. Rev..

[10] Gupta A., Behl T., Panichayupakaranan P. (2019). A Review of Phytochemistry and Pharmacology Profile of *Juglans regia*. Obes. Med..

[11] Zhang J., Zhang J., Zhao C., Sui H., Li C.F., Zhong L., Zhou Q., Bai Y., An S., Du X. (2022). Green Walnut Husk Extracts Proliferation and Migration in Gastric Cancer. J. Cancer.

[12] Alshatwi A.A., Hasan T.N., Shafi G., Syed N.A., Al-Assaf A.H., Alamri M.S., Al-Khalifa A.S. (2012). Validation of the Antiproliferative Effects of Organic Extracts from the Green Husk of *Juglans regia* L. on PC-3 Human Prostate Cancer Cells by Assessment of Apoptosis-Related Genes. Evid. Based Complement. Alternat. Med..

[13] Beiki T., Najafpour G.D., Hosseini M. (2018). Evaluation of Antimicrobial and Dyeing Properties of Walnut ( *Juglans regia* L.) Green Husk Extract for Cosmetics. Color. Technol..

[14] Masek A., Latos-Brozio M., Chrzescijanska E., Podsedek A. (2019). Polyphenolic Profile and Antioxidant Activity of *Juglans regia* L. Leaves and Husk Extracts. Forests.

[15] Zeb A., Ali G., Al-Babili S. (2024). Comparative UHPLC-MS/MS-Based Untargeted Metabolomics Analysis, Antioxidant, and Anti-Diabetic Activities of Six Walnut Cultivars. Food Biosci..

[16] Santos A., Barros L., Calhelha R.C., Dueñas M., Carvalho A.M., Santos-Buelga C., Ferreira I.C.F.R. (2013). Leaves and Decoction of *Juglans regia* L.: Different Performances Regarding Bioactive Compounds and in Vitro Antioxidant and Antitumor Effects. Ind. Crops Prod..

[17] Sheng F., Hu B., Jin Q., Wang J., Wu C., Luo Z. (2021). The Analysis of Phenolic Compounds in Walnut Husk and Pellicle by UPLC-Q-Orbitrap HRMS and HPLC. Molecules.

[18] Savić I.M., Savić Gajić I.M. (2025). Extraction and Characterization of Antioxidants and Cellulose from Green Walnut Husks. Foods.

[19] Rajković K.M., Stanković M., Markićević M., Zavišić G., Vranješ-Đurić S., Janković D., Obradović Z., Stanković D. (2024). Chemical Composition and Protective Possibilities of Juglans Nigra Leaves and Green Husks Extracts: DNA Binding and Micronucleus Assay in Human Lymphocytes. Plants.

[20] Du H., Li C., Wen Y., Tu Y., Zhong Y., Yuan Z., Li Y., Liang B. (2014). Secondary Metabolites from Pericarp of *Juglans regia*. Biochem. Syst. Ecol..

[21] Gawlik-Dziki U., Durak A., Pecio Ł., Kowalska I. (2014). Nutraceutical Potential of Tinctures from Fruits, Green Husks, and Leaves of *Juglans regia* L.. Sci. World J..

[22] Barekat S., Nasirpour A., Keramat J., Dinari M., Meziane-Kaci M., Paris C., Desobry S. (2022). Phytochemical Composition, Antimicrobial, Anticancer Properties, and Antioxidant Potential of Green Husk from Several Walnut Varieties (*Juglans regia* L.). Antioxidants.

[23] Wu S., Pang Y., He Y., Zhang X., Peng L., Guo J., Zeng J. (2021). A Comprehensive Review of Natural Products against Atopic Dermatitis: Flavonoids, Alkaloids, Terpenes, Glycosides and Other Compounds. Biomed. Pharmacother..

[24] Zhao M.-H., Jiang Z.-T., Liu T., Li R. (2014). Flavonoids in *Juglans regia* L. Leaves and Evaluation of *In Vitro* Antioxidant Activity via Intracellular and Chemical Methods. Sci. World J..

[25] Liu R., Zhao Z., Dai S., Che X., Liu W. (2019). Identification and Quantification of Bioactive Compounds in *Diaphragma Juglandis* Fructus by UHPLC-Q-Orbitrap HRMS and UHPLC-MS/MS. J. Agric. Food Chem..

[26] Xi M., Hou Y., Cai Y., Shen H., Ao J., Li M., Wang J., Luo A. (2023). Antioxidant and Antimicrobial Characteristics of Ethyl Acetate Polar Fractions from Walnut Green Husk. J. Food Sci..

[27] Chemineau P., Lainé A.L., Gennetay D., Porte C., Chesneau D., Laclie C., Goudet G., Meunier M., Delmas M., Greil M.L. (2025). The Walnut Tree as a Source of Progesterone for Reproductive Control in Goats. Animal.

[28] Cirone M., D’Orazi G. (2022). NRF2 in Cancer: Cross-Talk with Oncogenic Pathways and Involvement in Gammaherpesvirus-Driven Carcinogenesis. Int. J. Mol. Sci..

[29] Wang J., Kaplan M.H., Yang K. (2020). ResTORing Barrier Function in the Skin. J. Allergy Clin. Immunol..

[30] Goleva E., Berdyshev E., Leung D.Y.M. (2019). Epithelial Barrier Repair and Prevention of Allergy. J. Clin. Investig..

[31] Ding X., Bloch W., Iden S., Rüegg M.A., Hall M.N., Leptin M., Partridge L., Eming S.A. (2016). mTORC1 and mTORC2 Regulate Skin Morphogenesis and Epidermal Barrier Formation. Nat. Commun..

[32] Vieira V., Pereira C., Abreu R.M.V., Calhelha R.C., Alves M.J., Coutinho J.A.P., Ferreira O., Barros L., Ferreira I.C.F.R. (2020). Hydroethanolic Extract of *Juglans regia* L. Green Husks: A Source of Bioactive Phytochemicals. Food Chem. Toxicol..

[33] Mabasa X.E., Mathomu L.M., Madala N.E., Musie E.M., Sigidi M.T. (2021). Molecular Spectroscopic (FTIR and UV-Vis) and Hyphenated Chromatographic (UHPLC-qTOF-MS) Analysis and In Vitro Bioactivities of the Momordica Balsamina Leaf Extract. Biochem. Res. Int..

[34] He Y., Mao S., Zhao Y., Yang J. (2025). Research Advances in the Synthesis, Metabolism, and Function of Chlorogenic Acid. Foods.

[35] Wang L., Pan X., Jiang L., Chu Y., Gao S., Jiang X., Zhang Y., Chen Y., Luo S., Peng C. (2022). The Biological Activity Mechanism of Chlorogenic Acid and Its Applications in Food Industry: A Review. Front. Nutr..

[36] Habtemariam S. (2019). Introduction to Plant Secondary Metabolites—From Biosynthesis to Chemistry and Antidiabetic Action. Medicinal Foods as Potential Therapies for Type-2 Diabetes and Associated Diseases.

[37] Gnonlonfin G.J.B., Sanni A., Brimer L. (2012). Review Scopoletin—A Coumarin Phytoalexin with Medicinal Properties. Crit. Rev. Plant Sci..

[38] Jung S.-H., Heo H.-Y., Choe J.-W., Kim J., Lee K. (2021). Anti-Melanogenic Properties of Velutin and Its Analogs. Molecules.

[39] Xie C., Kang J., Li Z., Schauss A.G., Badger T.M., Nagarajan S., Wu T., Wu X. (2012). The Açaí Flavonoid Velutin Is a Potent Anti-Inflammatory Agent: Blockade of LPS-Mediated TNF-α and IL-6 Production through Inhibiting NF-κB Activation and MAPK Pathway. J. Nutr. Biochem..

[40] Akbari V., Jamei R., Heidari R., Esfahlan A.J. (2012). Antiradical Activity of Different Parts of Walnut (*Juglans regia* L.) Fruit as a Function of Genotype. Food Chem..

[41] (2022). Biology of Quinoline and Quinazoline Alkaloids. The Alkaloids: Chemistry and Biology.

[42] Stampar F., Solar A., Hudina M., Veberic R., Colaric M. (2006). Traditional Walnut Liqueur–Cocktail of Phenolics. Food Chem..

[43] Cosmulescu S., Trandafir I., Nour V., Ionica M., Tutulescu F. (2014). Phenolics Content, Antioxidant Activity and Color of Green Walnut Extracts for Preparing Walnut Liquor. Not. Bot. Horti Agrobot..

[44] Jakopic J., Solar A., Colaric M., Hudina M., Veberic R., Stampar F. (2008). The Influence of Ethanol Concentration on Content of Total and Individual Phenolics in Walnut Alcoholic Drink. Acta Aliment..

[45] Oliveira I., Sousa A., Ferreira I.C.F.R., Bento A., Estevinho L., Pereira J.A. (2008). Total Phenols, Antioxidant Potential and Antimicrobial Activity of Walnut (*Juglans regia* L.) Green Husks. Food Chem. Toxicol..

[46] Fernández-Agulló A., Pereira E., Freire M.S., Valentão P., Andrade P.B., González-Álvarez J., Pereira J.A. (2013). Influence of Solvent on the Antioxidant and Antimicrobial Properties of Walnut (*Juglans regia* L.) Green Husk Extracts. Ind. Crops Prod..

[47] Gagliardi S., Mitruccio M., Di Corato R., Romano R., Aloisi A., Rinaldi R., Alifano P., Guerra F., Bucci C. (2024). Defects of Mitochondria-Lysosomes Communication Induce Secretion of Mitochondria-Derived Vesicles and Drive Chemoresistance in Ovarian Cancer Cells. Cell Commun. Signal..

[48] Chacko B.K., Kramer P.A., Ravi S., Benavides G.A., Mitchell T., Dranka B.P., Ferrick D., Singal A.K., Ballinger S.W., Bailey S.M. (2014). The Bioenergetic Health Index: A New Concept in Mitochondrial Translational Research. Clin. Sci..

[49] Sreedhar A., Aguilera-Aguirre L., Singh K.K. (2020). Mitochondria in Skin Health, Aging, and Disease. Cell Death Dis..

[50] Martic I., Papaccio F., Bellei B., Cavinato M. (2023). Mitochondrial Dynamics and Metabolism across Skin Cells: Implications for Skin Homeostasis and Aging. Front. Physiol..

[51] Gaetano V., Gagliardi A., Giuliano E., Longo E., Cosco D. (2025). Chitosan Nanoparticles Loaded with Polyphenols for Cosmeceutical Applications: A State-of-the-Art Review. Pharmaceutics.

