# Supplementary material for: Phytochemistry and Biological Effects of the Juglans regia “Sorrento” Walnut Husk Extract on Human Keratinocyte Cells
Source: Antioxidants (Basel). 2025 Nov 21;14(12):1385. doi: 10.3390/antiox14121385 (PMC12729365; doi:10.3390/antiox14121385)
Supplement: Supplementary file 1 [file antioxidants-14-01385-s001.zip › antioxidants-3962859-supplementary.pdf]

Phytochemistry and Biological Effects of the *Juglans regia* "*Sorrento*" Walnut Husks Extract on Human Keratinocyte Cells

Giulia Vergine [1,2,†], Michela Ottolini [2,†], Giuseppe E. De Benedetto [3], Simona Bettini [4], Francesca Baldassarre [4,5,*], Daniele Vergara [2,*] and Giuseppe Ciccarella [4,5]

[1] National Interuniversity Consortium of Materials Science and Technology (INSTM), Via G. Giusti 9, 50121 Florence, Italy; giulia.vergine@unisalento.it

[2] Department of Biological and Environmental Sciences and Technologies (DiSTeBA), University of Salento, 73100 Lecce, Italy; michela.ottolini@unisalento.it

[3] Department of Cultural Heritage, University of Salento, Via D. Birago 64, 73100 Lecce, Italy; giuseppe.debenedetto@unisalento.it

[4] Department of Biological and Environmental Sciences and Technologies (DiSTeBA), University of Salento & UdR INSTM Salento, 73100 Lecce, Italy; simona.bettini@unisalento.it (S.B.); giuseppe.ciccarella@unisalento.it (G.C.)

[5] Institute of Nanotechnology, Consiglio Nazionale Delle Ricerche, CNR NANOTEC, Via Monteroni, 73100 Lecce, Italy

* Correspondence: francesca.baldassarre@unisalento.it (F.B.); daniele.vergara@unisalento.it (D.V.); Tel.: +39-0832-299469 (F.B.)

† These authors contributed equally to this work.

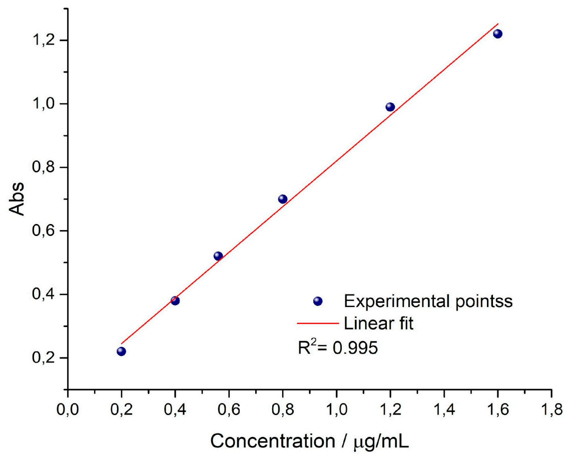

Figure S1. Gallic acid calibration curves for Folin –Ciocalteu colorimetric assay.

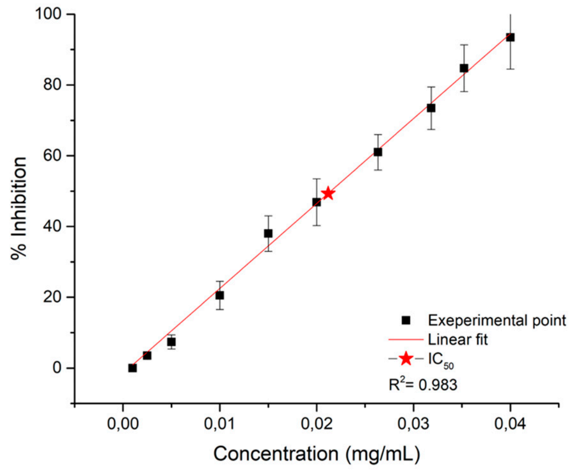

Figure S2. IC_{50} values of antioxidant activities monitored by ABTS assay. Linear regression curve of ABTS radical scavenging activity of WHE extracts; the red star indicates the calculated IC_{50} value.

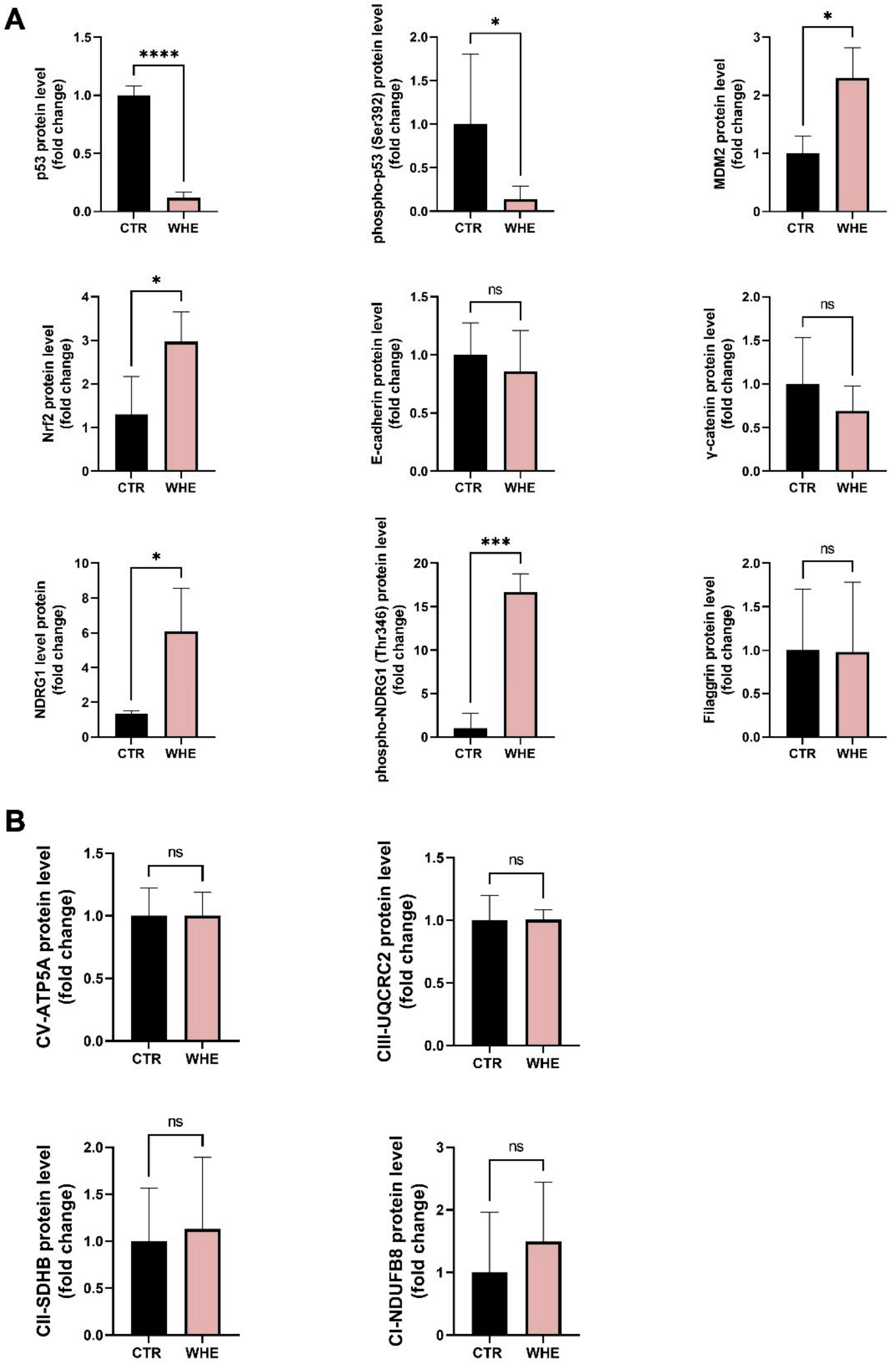

Figure S3. Densitometric quantification of all proteins analyzed through western blot. Histograms comparing protein expression levels in **(A)** p53, phospho-p53, MDM2, Nrf2, E-cadherin, γ-catenin, NDRG1, phospho-NDRG1, and filaggrin; and **(B)** OXPHOS complexes. Protein levels are plotted on a linear scale and expressed as fold change relative to control. Each bar represents the mean ± SD of at least three independent experiments, with values normalized to GAPDH. Graphs were generated using GraphPad Prism (version 9.5). Statistical significance was evaluated by *t*-test between CTR and WHE. (ns) not significant; *()* $p < 0.05$; (**) $p < 0.005$; (***) $p < 0.001$.

Classification	Compound Identification	Ione mode	(m/z)	Δ ppm*	Formula
**Beta Hydroxy Acids**	L-Malic Acid	$[M-H]^-$	133	1,073	$C_4H_6O_5$
**Butenolides**	Ascorbic Acid	$[M-H]^-$	175,02	1,099	$C_6H_8O_6$
**Flavanols**	(-)-Epicatechin	$[M-H]^+$	291,09	-0,818	$C_{15}H_{14}O_6$
	Epicatechin Gallate	$[M-H]^+$	443,1	-0,725	$C_{22}H_{18}O_{10}$
	Catechin	$[M-H]^-$	289,071	1,196	$C_{15}H_{14}O_6$
	Catechin Gallate	$[M-H]^-$	441,082	1,29	$C_{22}H_{18}O_{10}$
	Epiaxifolin	$[M-H]^+$	305,065	-0,814	$C_{15}H_{12}O_7$
	Taxifolin	$[M-H]^+$	305,065	-0,814	$C_{15}H_{12}O_7$
**Flavanones**	Eriodictyol	$[M-H]^+$	289,07	-0,818	$C_{15}H_{12}O_6$
	Naringenin	$[M-H]^+$	273,08	-0,823	$C_{15}H_{12}O_5$
	Naringenin chalcone	$[M-H]^-$	271,061	1,192	$C_{15}H_{12}O_5$
	Sternbin	$[M-H]^+$	303,09	-0,808	$C_{16}H_{14}O_6$
**Flavonoid-3-O-glycosides**	Hyperoside	$[M-H]^+$	465,1	-0,727	$C_{21}H_{20}O_{12}$
	Quercetin-3-Arabinoside	$[M-H]^+$	435,09	-0,742	$C_{20}H_{18}O_{11}$
	Quercetin 3-rhamnoside	$[M-H]^-$	447,093	1,284	$C_{21}H_{20}O_{11}$
	Kaempferol 3-O-glucoside	$[M-H]^+$	449,107	-0,731	$C_{21}H_{20}O_{11}$
	Myricitrin	$[M-H]^-$	463,09	1,288	$C_{21}H_{20}O_{12}$
	Astilbin	$[M-H]^-$	449,11	1,284	$C_{21}H_{22}O_{11}$
	Quercetin-3-o-beta-d-xylopyranoside	$[M-H]^-$	433,077	1,273	$C_{20}H_{18}O_{11}$
**Flavonols**	(+/-)-Dihydrokaempferol	$[M-H]^-$	287,06	1,196	$C_{15}H_{12}O_6$
	Quercetin	$[M-H]^-$	301,04	1,2	$C_{15}H_{10}O_7$
**Other Flavonoids**	2 beta-D-glucopyranosyl Phloretin	$[M-H]^+$	275,09	-0,823	$C_{15}H_{14}O_5$
	Phloretin	$[M-H]^-$	273,076	1,192	$C_{15}H_{14}O_5$
	Luteolin 3',4'-Dimethyl Ether	$[M-H]^-$	313,07	1,218	$C_{17}H_{14}O_6$
	Velutin	$[M-H]^+$	315,09	-0,797	$C_{17}H_{14}O_6$

**Hexoses**	Mannose	$[M-H]^-$	179,06	1,1	$C_6H_{12}O_6$
**Hydrolyzable Tannins**	Ellagic Acid	$[M-H]^-$	301	1,194	$C_{14}H_6O_8$
**Hydroquinolones**	2-Hydroxyquinoline	$[M-H]^-$	144,05	1,113	C_9H_7NO
**Phenolic acids**	Shikimic Acid	$[M-H]^-$	173,05	1,105	$C_7H_{10}O_5$
	Dihydrocoumaroyl Hexoside	$[M-H]^-$	327,108	1,206	$C_{15}H_{20}O_8$
	Esculetin	$[M-H]^-$	177,02	1,122	$C_9H_6O_4$
	Scopoletin	$[M-H]^+$	193,05	-0,881	$C_{10}H_8O_4$
	Quinic Acid	$[M-H]^-$	191,06	1,11	$C_7H_{12}O_6$
**Phenolic acids (hydroxybenzoic acids)**	Gallic Acid isomer	$[M-H]^-$	169,01	1,105	$C_7H_6O_5$
	Gallic Acid hexoside	$[M-H]^-$	331,068	1,105	C13H16O10
	Pyrocatechuic Acid	$[M-H]^-$	153,02	1,101	$C_7H_6O_4$
	Syringic Acid	$[M-H]^-$	197,05	1,127	$C_9H_{10}O_5$
**Phenolic acids (hydroxycinnamic acid)**	Chlorogenic Acid	$[M-H]^+$	355,1	-0,794	$C_{16}H_{18}O_9$
	3-p-Coumaroylquinic Acid	$[M-H]^-$	337,09	1,216	$C_{16}H_{18}O_8$
	Neochlorogenic Acid	$[M-H]^-$	353,09	1,221	$C_{16}H_{18}O_9$
	5-O-Feruloylquinic Acid	$[M-H]^-$	367,1	1,232	$C_{17}H_{20}O_9$
	Ferulic Acid	$[M-H]^+$	195,07	-0,881	$C_{10}H_{10}O_4$
	4-Hydroxy-3-methoxycinnamic acid isomer	$[M-H]^-$	193,050	1,133	$C_{10}H_{10}O_4$
**Secondary Alcohols**	D-Pantothenic Acid	$[M-H]^-$	218,1	1,132	$C_9H_{17}NO_5$
**Tannins**	1,6-Digalloyl-Beta-D-Glucopyranose	$[M-H]^-$	483,08	1,286	$C_{20}H_{20}O_{14}$
	1,2,3,6-Tetragalloylglucose	$[M-H]^-$	787,1	1,473	$C_{34}H_{28}O_{22}$
	[3,4,5-Trihydroxy-6-[[(E)-3-(4-Hydroxyphenyl)Prop-2-Enoyl]Oxymethyl]Oxan-2-Yl] 3,4,5-Trihydroxybenzoate	$[M-H]^-$	477,1	1,299	$C_{22}H_{22}O_{12}$
**Tricarboxylic Acids and Derivatives**	Citric Acid	$[M-H]^-$	191,01	1,104	$C_6H_8O_7$
**Tropolones**	Pyrogallin	$[M-H]^-$	203,04	1,144	$C_{11}H_8O_4$

**Other compounds**	Adenine	$[M-H]^-$	134,05	1,079	$C_5H_5N_5$
	Arabinofuranosyluracil	$[M-H]^-$	243,06	1,139	$C_9H_{12}N_2O_6$
	Citramalic Acid	$[M-H]^-$	147,03	1,084	$C_5H_8O_5$
	D-Glyceric Acid	$[M-H]^-$	105,02	1,057	$C_3H_6O_4$
	Hyacinthacine	$[M-H]^+$	220,117	-0,883	$C_9H_{17}NO_5$
	LPE 16:0	$[M-H]^-$	452,28	1,272	$C_{21}H_{44}NO_7P$
	LPE 18:1	$[M-H]^-$	478,29	1,294	$C_{23}H_{46}NO_7P$
	LPE 18:2	$[M-H]^-$	476,28	1,293	$C_{23}H_{44}NO_7P$
	LPE 18:3	$[M-H]^-$	474,26	1,293	$C_{23}H_{42}NO_7P$
	LPI 18:2	$[M-H]^-$	595,29	1,356	$C_{27}H_{49}O_{12}P$
	Octopine	$[M-H]^+$	247,14	-0,876	$C_9H_{18}N_4O_4$
	Progesterone	$[M-H]^+$	315,23	-0,769	$C_{21}H_{30}O_2$
	Sayaendoside	$[M-H]^-$	415,16	1,259	$C_{19}H_{28}O_{10}$
	Tryptophan	$[M-H]^+$	205,1	-0,872	$C_{11}H_{12}N_2O_2$
	Tyramine	[M−H]+	138,09	-0,912	$C_8H_{11}NO$
	Vanillylmandelic Acid	$[M-H]^-$	197,05	1,127	$C_9H_{10}O_5$

Table S1. WHE compounds identified by UHPLC–Q-Orbitrap HRMS based on their chemical classification.
